# EHFPI: a database and analysis resource of essential host factors for pathogenic infection

**DOI:** 10.1093/nar/gku1086

**Published:** 2014-11-20

**Authors:** Yang Liu, Dafei Xie, Lu Han, Hui Bai, Fei Li, Shengqi Wang, Xiaochen Bo

**Affiliations:** 1 Department of Biotechnology, Beijing Institute of Radiation Medicine, Beijing 100850, P.R.China; 2 No. 451 Hospital of Chinese People's Liberation Army, Xi'an 710054, China

## Abstract

High-throughput screening and computational technology has greatly changed the face of microbiology in better understanding pathogen–host interactions. Genome-wide RNA interference (RNAi) screens have given rise to a new class of host genes designated as Essential Host Factors (EHFs), whose knockdown effects significantly influence pathogenic infections. Therefore, we present the first release of a manually-curated bioinformatics database and analysis resource EHFPI (Essential Host Factors for Pathogenic Infection, http://biotech.bmi.ac.cn/ehfpi). EHFPI captures detailed article, screen, pathogen and phenotype annotation information for a total of 4634 EHF genes of 25 clinically important pathogenic species. Notably, EHFPI also provides six powerful and data-integrative analysis tools, i.e. EHF Overlap Analysis, EHF-pathogen Network Analysis, Gene Enrichment Analysis, Pathogen Interacting Proteins (PIPs) Analysis, Drug Target Analysis and GWAS Candidate Gene Analysis, which advance the comprehensive understanding of the biological roles of EHF genes, as in diverse perspectives of protein–protein interaction network, drug targets and diseases/traits. The EHFPI web interface provides appropriate tools that allow efficient query of EHF data and visualization of custom-made analysis results. EHFPI data and tools shall keep available without charge and serve the microbiology, biomedicine and pharmaceutics research communities, to finally facilitate the development of diagnostics, prophylactics and therapeutics for human pathogens.

## INTRODUCTION

Infectious diseases result in millions of deaths each year. Many efforts have been made annually to better understand how pathogens interplay with their hosts and to identify potential targets for developing antimicrobial therapeutics. High-throughput RNA interference (RNAi) screening technologies have in recent years facilitated the research of pathogen–host interactions. These systematic, genome-wide loss-of-function experiments can be used to identify host genes that increase or inhibit pathogenic infections. Typically, the validated host genes, whose loss have totally or partially inhibited pathogenic infection but not cell viability, are designated as Essential Host Factors (EHFs).

Ever since 2002, genome-wide RNAi screens for dozens of clinically important priority human bacterial and viral pathogens have been carried out (1). However, the vast majority of experimental results that have been gained in the field are hidden in the prose of scientific literature. Moreover, for a specific pathogen species or strain, each published study nominates hundreds of genes as EHFs, whereas these EHF data generated by different labs using different RNAi systems present low repeating rate (2–4). Thus, categorization of these amounting literature-based EHF data in an unbiased way is a great challenge that may provide a powerful framework for future data curation. Meanwhile, these EHF records can be enhanced through integration with additional knowledge on screen information and system analysis.

With foresight into the above important aspects of such information, we collected all related literatures from peer-reviewed publications and developed an open, integrated online resource for EHF data about human pathogens. The database and analysis resource of EHFs for Pathogenic Infection (EHFPI, http://biotech.bmi.ac.cn/ehfpi) serves as a publicly accessible repository for manually curated EHFs categorized as confirmed hits and primary hits, which show significant effects on single pathogen infection when knockdown *in vitro* (Supplementary Figure S1). EHFPI is unique among other RNAi screen-based databases in that it specifically contains a wealth of curated and integrated EHF information for a large number of bacterial, viral and fungal families that are pathogenic to humans. This is in contrast with the Database of Essential Genes (DEG, http://www.essentialgene.org/) (5), which provides information on a particular group of genes indispensable for the survival of an organism, or the *Drosophila* RNAi Screening Center (DRSC, http://www.flyrnai.org) (6) and the GenomeRNAi database (http://www.genomernai.org) (7), which focuses on a particular screen system or makes available the screen results by directly extracting RNAi phenotype information from the literature. Most importantly, EHFPI also integrates a set of analysis tools suited to elucidate network relationships, decipher biological importance and generate hypotheses to be experimentally tested for pathogen–host interaction, drug discovery, and infection-disease association. With the necessary EHF data, bioinformatics tools and workflows, EHFPI enhances the understanding of host-pathogen interplay and promotes the development of diagnostics, prophylactics and therapeutics for confronting human pathogens.

## DATA SOURCE

**Figure 1.**
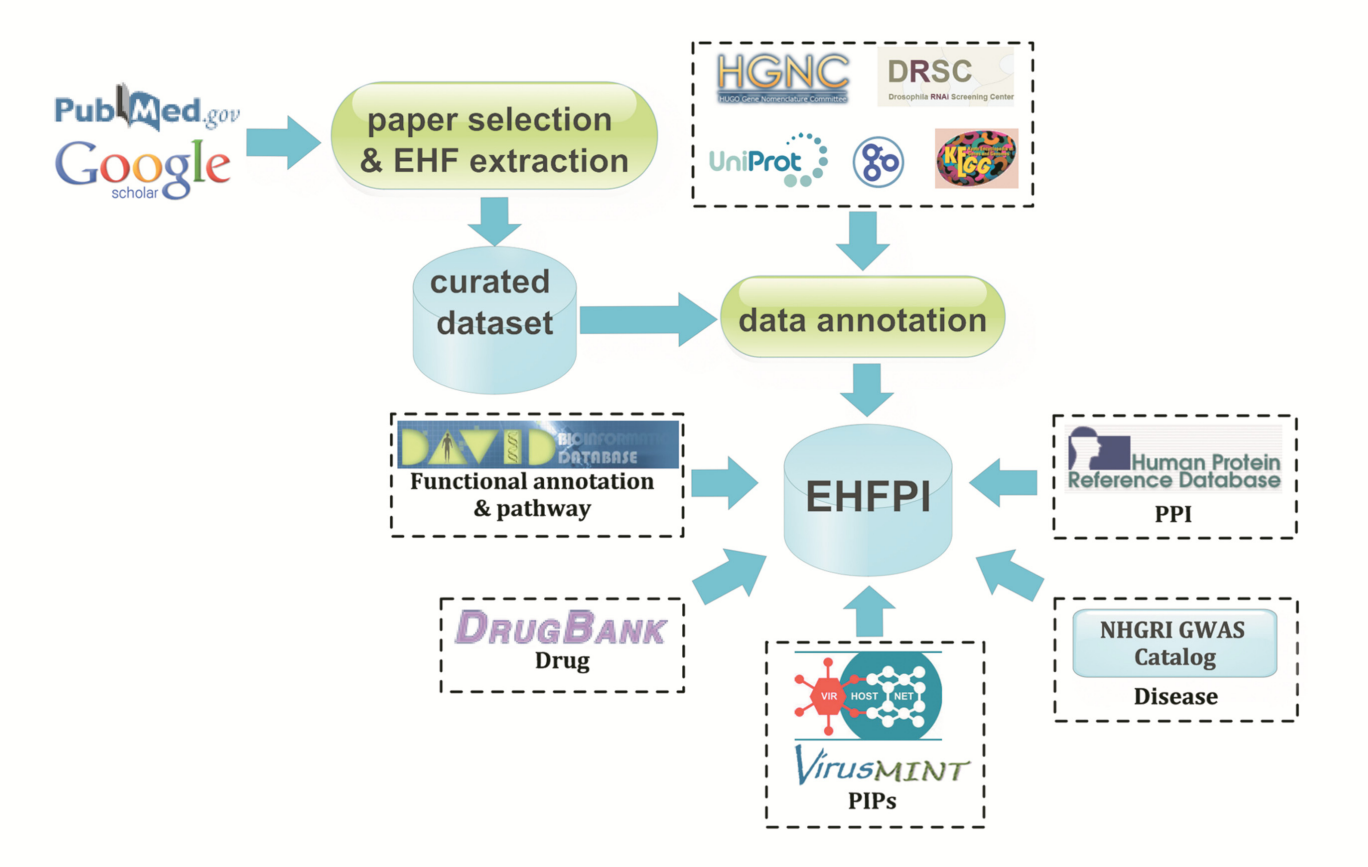
Data source of EHFPI. EHF related literatures were obtained from peer-viewd publications through search on PubMed and Google Scholar. The curated EHF gene datasets for different pathogens were further complemented by annotations from external databases, e.g. HGNC, *Drosophila* RNAi Screening Center, UniProt, Gene Ontology and KEGG. The drug target information was collected from DrugBank. Information on disease/traits associated single nucleotide polymorphisms in GWAS candidate gene(s) was from NHGRI GWAS Catalog. Gene function and pathway information was from DAVID. The information on pathogen interacting host proteins was from VirHostNet and VirusMINT. Human protein–protein interaction (PPI) information was from Human Protein Reference Database (HPRD).

The EHF data and related information were collected and extracted from open published literatures. Before submitting to EHFPI database, all the EHF data were manually curated and verified by domain experts. The EHF related annotation and association analysis bioinformatics resources have also been integrated into EHFPI (Figure 1).

### Manual curation

The EHFPI database contains EHFs supported by strong experimental evidence from open-published studies. The major source to obtain these is to search literature on PubMed and Google Scholar, using the keywords ‘genome-wide RNAi screen OR RNAi screen’. Literatures reporting screens for identifying host genes essential for pathogenic infections were specifically extracted by reviewing the article. Once the screens were verified, EHF data and related information on article, screen, pathogen and phenotype(s) were manually extracted from original scientific literature by domain experts.

Despite the differences in screen system, reagents and scope, a typical genome-wide RNAi screen is a multistep standard procedure that mainly includes (Supplementary Figure S1): (i) the primary screen that offers preliminary genes designated as ‘hits’, if their influence on infection is scored above a threshold level, (ii) the secondary or tertiary screen that repeats the RNAi screen only on (a portion of) hit genes and generates candidate genes designated as ‘confirmed hits’, complemented with polymerase chain reaction validation for confirmed RNAi effects and cell viability test, and (iii) *in vitro* and/or *in vivo* function study on one or a few of the confirmed hit genes, often with top scores and designated as ‘primary hits’, to further address their essentiality and regulation mechanism in certain step of infection, e.g. entry, replication, or phagocytosis.

Therefore, instead of providing a copy of simplified original screen result lists that authors offer in the supplementary files, we extracted only confirmed hits and primary hits for a specific pathogen species/strain (confirmation were made through correspondance with authors when mismatches on reported numbers occur). We computationally combined the confirmed hits generated from individual screen using the same pathogen species/strain by removing the repeating items and designated the total non-redundant gene set of confirmed hits as EHFs for a specific pathogen type/species. To be noted, for the majority of genome-wide RNAi screens, the authors publish only results that show genes whose knockdown effects significantly inhibit infection. For the integrity of EHF data, we took account of EHF genes with phenotype of both increased and decreased infection when silenced *in vitro*, which are differentially categorized and notated in the database.

Up to the data revision of 30 September 2014, EHFPI contains 4634 EHF genes and related annotation information for 25 clinically important human pathogen types (including 10 bacterial species, 14 viral species and 1 fungal species), reported by 40 studies. The data now feature 41 genome-wide RNAi screens in *Homo sapiens* and 24 in *Drosophila*, with a total of 65 gene-phenotype associations (Supplementary Figure S2).

### Data annotation

MIARE recommendation to annotate RNAi screens (http://miare.sourceforge.net) has been widely acknowledged and applied in characterizing single RNAi experiment. However, most genome-wide RNAi screens use commercialized libraries and follow designated protocols, and screen annotations have been provided in a simple and summarized way rather than a compendium in a MIARE-compliant format. Thus, we have exploited generic items in the MIARE recommendation to annotate genome-wide RNAi screens, and the detailed annotations in EHFPI database follow in part the MIARE recommendation. Specifically, EHF data in EHFPI is organized and annotated as in four main categories, i.e. gene information, pathogen information, article information and screen information, which are interconnected with an interaction database entity (Supplementary Figure S3).

Of note, most genome-wide RNAi screens that identify EHFs for bacterial and fungal pathogens are performed in the *Drosophila* system, which provides a good entry-point into learning about human biology in confrontation of infection. For these *Drosophila* system based screens, the EHF data as in both ways of *Drosophila* genes symbols and human orthologous are provided and independently listed. To this end, we converted the *Drosophila* EHF genes to human orthologous, as referenced using the DRSC Integrative Ortholog Prediction Tool (DIOPT) (8). DIOPT integrates human, mouse, fly, worm, zebrafish and yeast ortholog predictions made by Ensembl Compara, HomoloGene, Inparanoid, Isobase, OMA, orthoMCL, Phylome, RoundUp and TreeFam. For a given *Drosophila* gene, DIOPT calculates a simple score indicating the number of tools that support the given orthologous gene-pair relationship, as well as a weighted score based on functional assessment using high quality GO molecular function annotation of all fly-human orthologous pairs predicted by each tool. And we select all 10 prediction tools and use the filter ‘return only best match when there is more than one match per input gene or protein’. Our treatment ensures that we retain the best matched gene-pair presented in EHFPI database, despite a few orthologous with low fidelity not included. Meanwhile, some human gene symbols in early literatures may be withdrawn now, or synonyms or previous names of an official gene symbol. To provide the EHF gene content of EHFPI in a standardized format and facilitate the following analysis, we convert all gene symbols according to HGNC (9) official gene symbols.

EHFPI database also integrates external resources to enrich these basic annotations. Each EHF gene and its gene product information are annotated with HGNC and Uniprot. Gene location and GO terms information are from BioMart (10). KEGG pathway id information is converted with ID Converter System (11). Whenever possible, entries from EHFPI are hyperlinked to NCBI Taxonomy database, NCBI Gene database, PubMed, Uniprot, Gene Ontology and other resources. Human PIPs validated by high-throughput protein interaction experiments have been collected and presented for each viral pathogen from public databases of VirusMINT (30) and VirHostNet (31). The human PPI network is constructed with PPI information from HPRD (32). All these data are integrated into a knowledge-based system using MySQL Database Management System.

### Data update

EHFPI database will incorporate newly published literatures and related EHF data quarterly. It also provides a data update mechanism that allows users to submit their own manually-curated EHFs via an excel format file containing necessary items as required in data curation. Once reviewed by an expert, the data submitted will be published in EHFPI.

## ANALYSIS TOOLS

**Figure 2.**
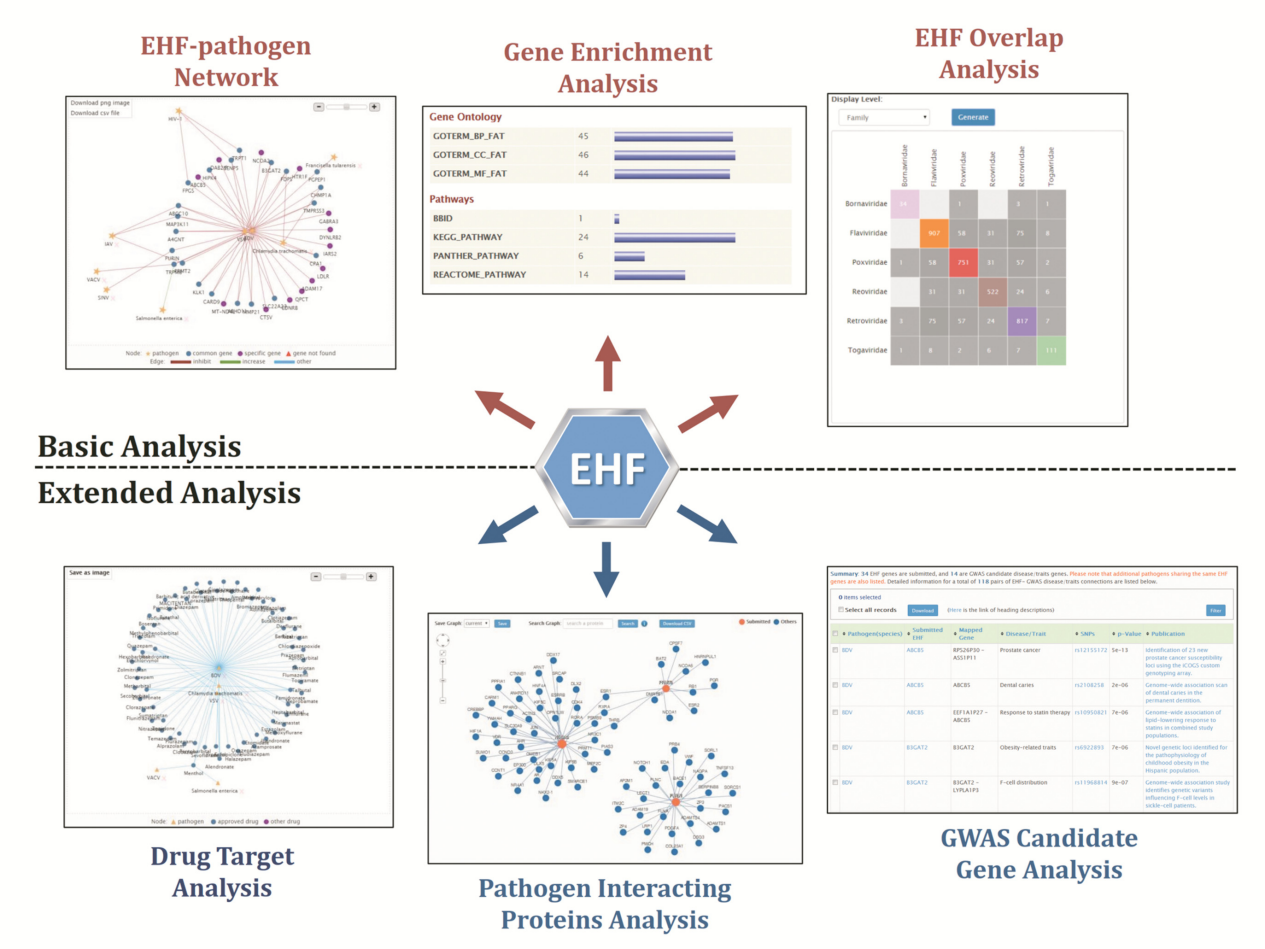
Six analysis tools of EHFPI to facilitate the in-depth understanding of EHF genes for pathogenic infection.

A flexible web interface that allows both basic and advanced SEARCH and BROWSE is designed and realized after thorough discussing and collaboration with experimental scientists (Supplementary results). Besides, the EHFPI database also provides six powerful and user-friendly analysis tools to facilitate the in-depth understanding of EHF genes (Figure 2):

- The ‘*EHF Overlap Analysis*’ is a custom-made tool to statistically compare EHFs from screens/publications reported by different labs or according to different pathogen taxonomy level, and to present the results in an annotated heat map representation.
- The ‘*EHF-pathogen Network Analysis*’ is a custom-made graphic tool designed to help researchers explore the relationships between EHFs and pathogens in an annotated network representation.
- The ‘*Gene Enrichment Analysis*’ is a basic and general analytical tool for functional annotation of EHF genes using DAVID (12) as external link (as well as other external tools such as GOEAST (13), PANTHER (14), Reactome (15), etc.). Users can explore the EHF genes as involved in different bioprocesses or signaling pathways.
- The ‘*Pathogen Interacting Proteins (PIPs) Analysis*’ is a specially designed tool for integrative understanding of EHFs as in the context of pathogen–host interactions. EHFs are interpreted by comparing with another type of important host factors for pathogenic infection, i.e. PIPs and also as in the human protein–protein interaction (PPI) network.
- The ‘*Drug Target Analysis*’ is a custom-made graphic tool designed to identify drugs that target EHFs and explore the potential of drug repositioning for host-directed anti-infection therapeutics in a bioinformatics way.
- The ‘*GWAS Candidate Gene Analysis*’ is a tool to identify the EHF genes associated with certain diseases/traits, which offers a new perspective to decipher infection-disease associations.

All these tools will be described in more details within context of the application case study described below.

## APPLICATION CASE STUDY

To showcase how these analysis tools are functionally fulfilled, we will use the EHF data of human Influenza A virus (IAV) to illustrate.

IAV is an important respiratory infectious virus with many subtypes or strains notable for their adaptability and resilience. Emergence of a new highly pathogenic IAV strain has always ended in a pandemic outbreak that resultes in millions of hospitalizations and deaths worldwide. However, highly effective vaccines and treatments are still in shortage. As an example use case, we will present and extend the knowledge on EHFs of IAV by providing to date the most extensive curated information as well as performing in-depth system analyses. Although this illustration is focused on IAV, it should be noted that similar tasks can be performed for other pathogen species to address other biological questions by combining the wealth of relevant data with the suite of bioinformatics tools integrated into EHFPI.

### Search for EHF records regarding IAV

To begin, a query is constructed for all EHF records regarding IAV strains isolated from humans to date. As to September 2014, this specific query returned six published studies reporting a total of 1213 non-redundant EHF genes with 3 phenotypes, which were experimentally identified from 10 screens using eight different IAV strains (Supplementary Figure S4).

### Multi-centric EHF overlap calculation and visualization

EHFPI allows multi-centric comparisons (i.e. pathogen taxonomy and screens) of EHF data and converts the results in a heat map representation that supports multi-order demonstrations (e.g. common gene number or pathogen name). Upon visual inspection of the comparison results based on screens/publications, it is obviously identified that the screen done by Shapira SD *et* *al*. (16) provides the most EHF genes (i.e. 616) for IAV, while the screen done by Su WC *et al*. (17) provides the least (i.e. 38) (Supplementary Figure S5A). By ordering the heat map display in way of Common Gene Number, it can be clearly seen that these two screens share the least EHF genes for IAV (i.e. 2), while the screens respectively done by Karlas A *et al*. (18) and König R et al. (19) share the most EHF genes for IAV (i.e. 27) (Supplementary Figure S5A). These different EHF sets of IAV are only overlapped by an extremely small portion (<16% in average), which reflects the experimental feature of genome-wide RNAi screens.

Further comparative analysis can be realized if the user tries to identify the overlap between different species of negative-sense single-stranded RNA viruses that include IAV. And by putting the heat map representation in the order of Pathogen Group, the results can be easily interpreted that IAV shares the most EHF genes with vesicular stomatitis virus (VSV) in the family *Rhabdoviridae*, and shares the least EHF genes with Borna disease virus in the family *Bornaviridae* (Supplementary Figure S5B). This special overlap analysis allows quick overview of EHF landscape in a statistical way for different pathogens under designated taxonomy level.

### EHF-pathogen network visualization and statistics

**Figure 3.**
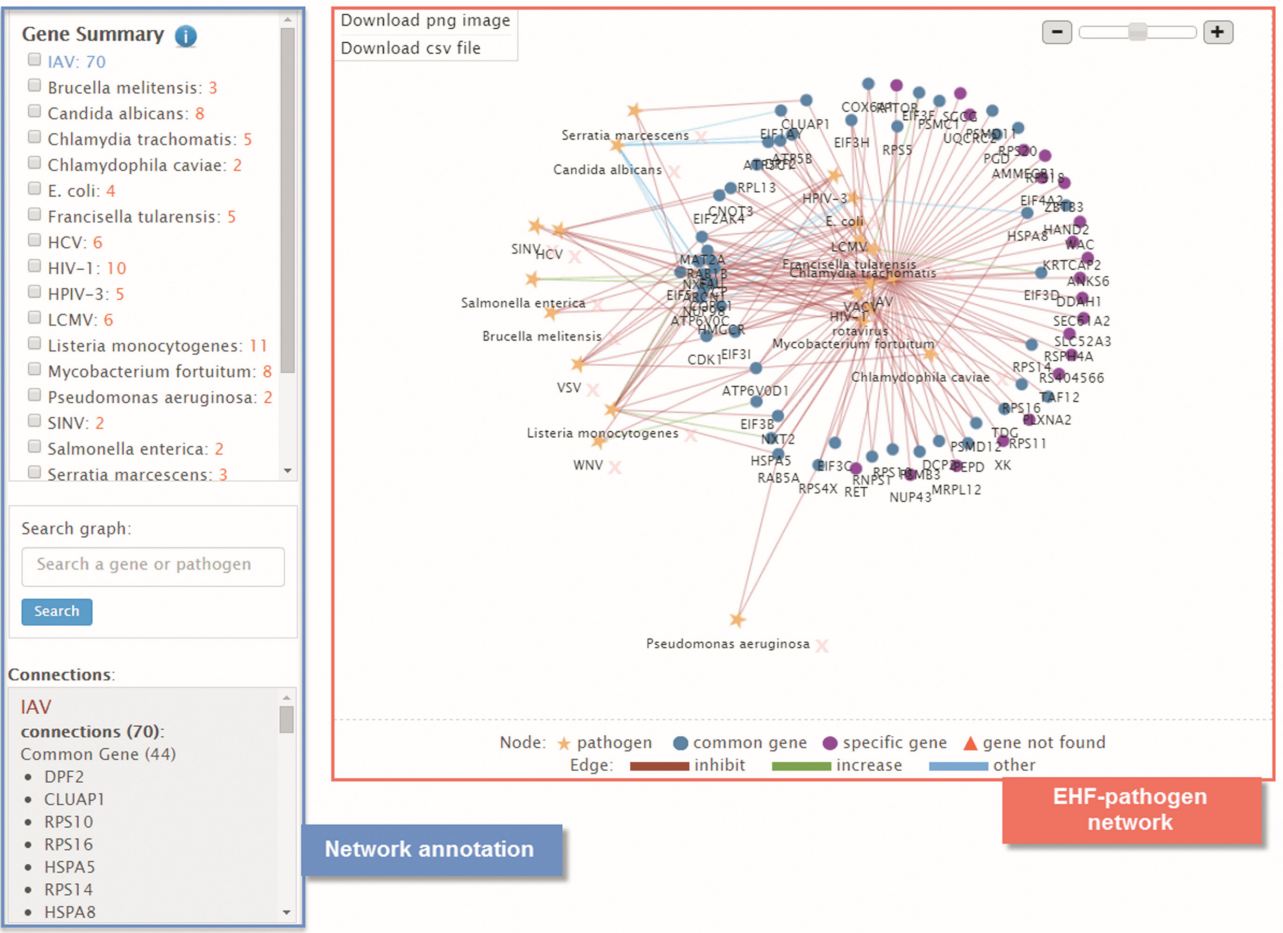
Visualization of the EHF-pathogen network for EHFs of IAV (for better illustration, only EHF data from one of the six articles reporting EHFs of IAV were used). EHF-pathogen network can be constructed on the EHFPI server using EHF records selected from a query result, an EHFPI guidance tree or uploaded to the system through a web interface. A graphic version of EHF-pathogen network can then be downloaded in png or csv format. For the current use case, an EHF-pathogen network is constructed with the desired EHF genes by choosing ‘IAV’ species in the guidance tree. Therefrom, the relationship of EHFs among pathogens can be viewed in the network graph viewer, which allows popover tooltips and user-driven editing of network ‘nodes’. This extremely facilitates information exploration and enables generation of an optimized layout of the elements (force-directed). Further within-network search can be performed using gene symbol(s) or pathogen name(s), with matched results highlighted in the viewer window. EHFs that are specific to a certain pathogen or shared by multiple pathogens are statistically summarized and listed in an independent display window at the same page. The customized graphic and statistics can then be downloaded or exported for enhanced interpretation and inclusion in publications.

EHF-pathogen network presents the global relationship of EHFs for designated pathogens in a visualized version and the connectivity of nodes (i.e. pathogens or EHF genes) reveals the associations of different pathogens on basis of shared EHFs. The graphic network and statistics show that IAV share different amount of EHF genes with a total of 24 different pathogens (Figure 3), including 13 viruses, 10 bacteria and 1 fungus. Specifically, IAV shares the most EHFs (i.e. 104) with human immunodeficiency virus type 1 (HIV-1) and the least EHFs (i.e. 2) with Dengue virus-2 (DENV-2). And the other top ranking pathogens that share EHFs with IAV include vaccinia virus (i.e. 97), hepatitis C virus (HCV) (i.e. 78), *Listeria monocytogenes* (i.e. 54), West Nile virus (i.e. 51), rotavirus (i.e. 50) and *Candida albicans* (i.e. 42).

Meanwhile, based on the network statistics provided in the csv file (Supplementary Table S1), 426 out of the 1213 EHFs of IAV are shared by two or more among the 24 pathogen types. And COPB1 is the EHF gene shared by most pathogen types, i.e. 14 pathogens including *Chlamydophila caviae*, *Escherichia coli*, *Francisella tularensis*, *L. monocytogenes*, *Mycobacterium fortuitum*, *Salmonella enterica*, *Serratia marcescens*, *C. albicans*, IAV, hepatitis B virus (HBV), human parainfluenza virus 3 (HPIV-3), lymphocytic choriomeningitis virus (LCMV), rotavirus and VSV. Moreover, ARCN1, COPA, COPG1, COPZ1 and COPB2 are the other five top ranking EHFs genes of IAV that share with more than 10 pathogen types. Of note, 787 out of the 1213 EHFs of IAV are specific to itself. This highly suggests that specific regulatory mechanisms underlie the host cellular response to IAV infection.

### EHFs in human protein–protein interaction network

PIPs are another important type of host factors that pathogens directly hijack for survival and replication. And PPI represents a pivotal aspect of protein function in numerous physiological and pathological processes with the aid of functional annotation. Human PPI network, where each point represents a protein and each line between them is an interaction, can reveal how complex molecular processes are activated in the cell. The PIP Analysis Tool is an automated workflow, specifically developed by the EHFPI team to assist researchers in taking advantage of the breadth of PIP data resource and accompanying the human PPI network.

**Figure 4.**
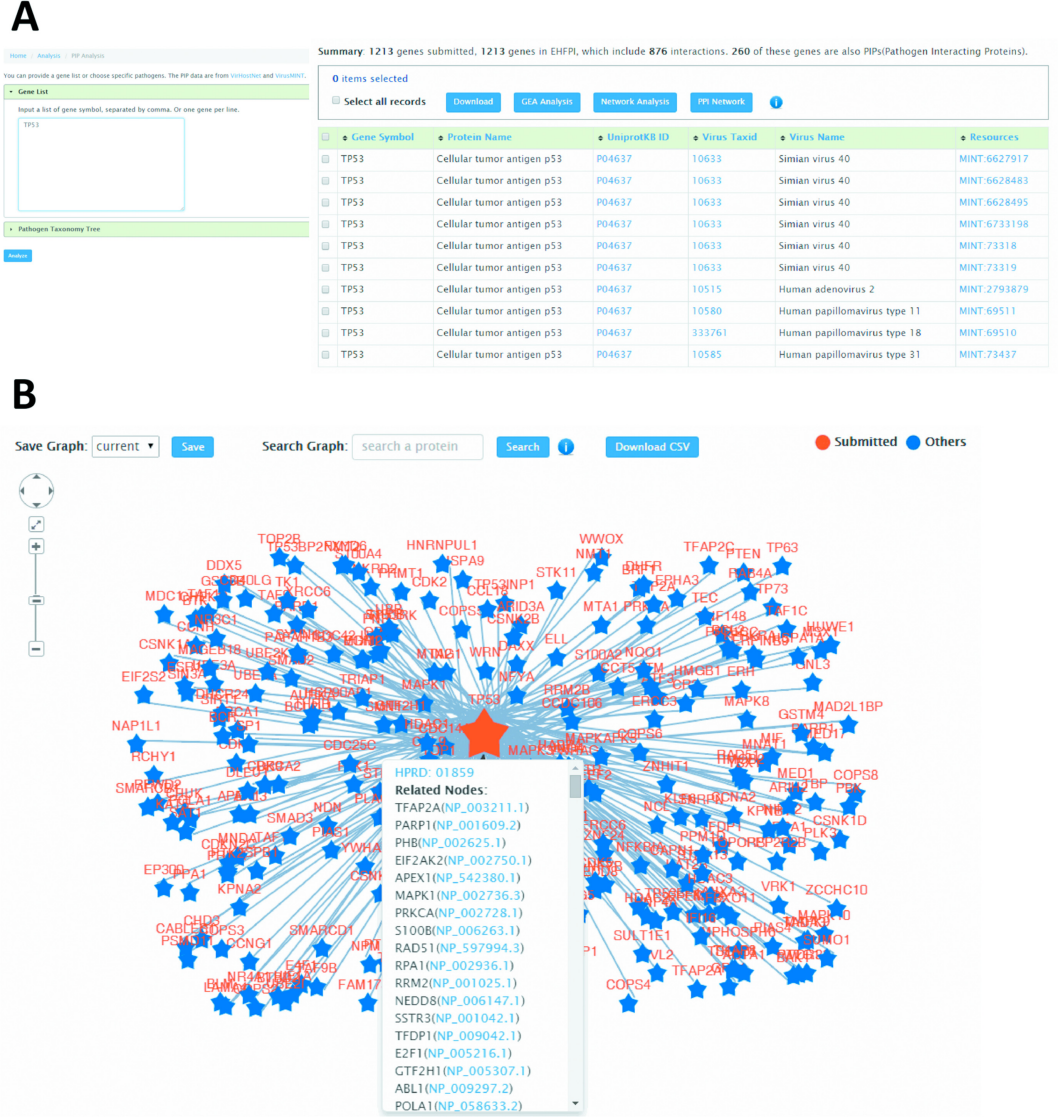
(**A**) List of pathogen interacting protein (PIP) analysis result for EHFs of IAV. (**B**) Partial illustration of the human PPI network for EHF gene TP53 of IAV. An automatically generated graph can be used to view the interactions and topology properties (hubs and bottlenecks) of EHFs in the human PPI network. And for each interaction, brief information on annotations and external links to supporting evidence are provided. Further within-network search can be performed using gene symbol(s), with matched results highlighted in the viewer window.

For the current use case, the PIP analysis identified 260 out of 1213 EHF genes (21.43%) for IAV as PIPs (Figure 4A), which means the large proportion of EHF gene products (78.57%) are not direct interacting proteins for IAV. Meanwhile, a total of 876 interactions were observed for the 260 EHF gene products that are also PIPs for IAV. Of note, the most highly interconnected EHF gene products for IAV include TP53 (i.e. 272 interactions, Figure 4B), EP300 (i.e. 211 interactions), GRB2 (i.e. 195 interactions) and PRKCA (i.e. 174 interactions), these basic topological properties of which can be easily summarized in the csv file for user download (Supplementary Table S2). This result implies that dysregulation of these EHF genes upon infection may perturb multiple host cell functions of biological essentiality and thereby affect the consequences where infection may lead to. Further, users can refer to the Search result on the start page of PIP analysis for detailed PIP resource information on interested EHF(s), e.g. TP53 (cellular tumor antigen p53, Figure 4A). This highly interacted EHF gene product of IAV, is also the PIP for eight other virus types, including HIV-1, HCV, Simian virus 40, Human adenovirus 1 and 2, Human papillomavirus (type 11, 16, 18 and 31), Bovine papillomavirus type 1, Alphapapillomavirus 7 and JC polyomavirus. Together, this comparative analysis of EHF and PIP provides insights into the functional understanding of EHFs at translational level.

### EHFs as potential targets in drug repositioning

Genome-wide RNAi screens for pathogenic infections have offered EHFs as a promising class of potential drug targets for antimicrobials. Instead of high-throughput screening, the DRUG TARGET tool of EHFPI represents a bioinformatics way to identify the FDA-approved drugs and other compounds (experimental, nutraceutical, investigational, and withdrawn) that target EHF gene products based on experimentally verified drug target information from DrugBank 4.1 (20).

**Figure 5.**
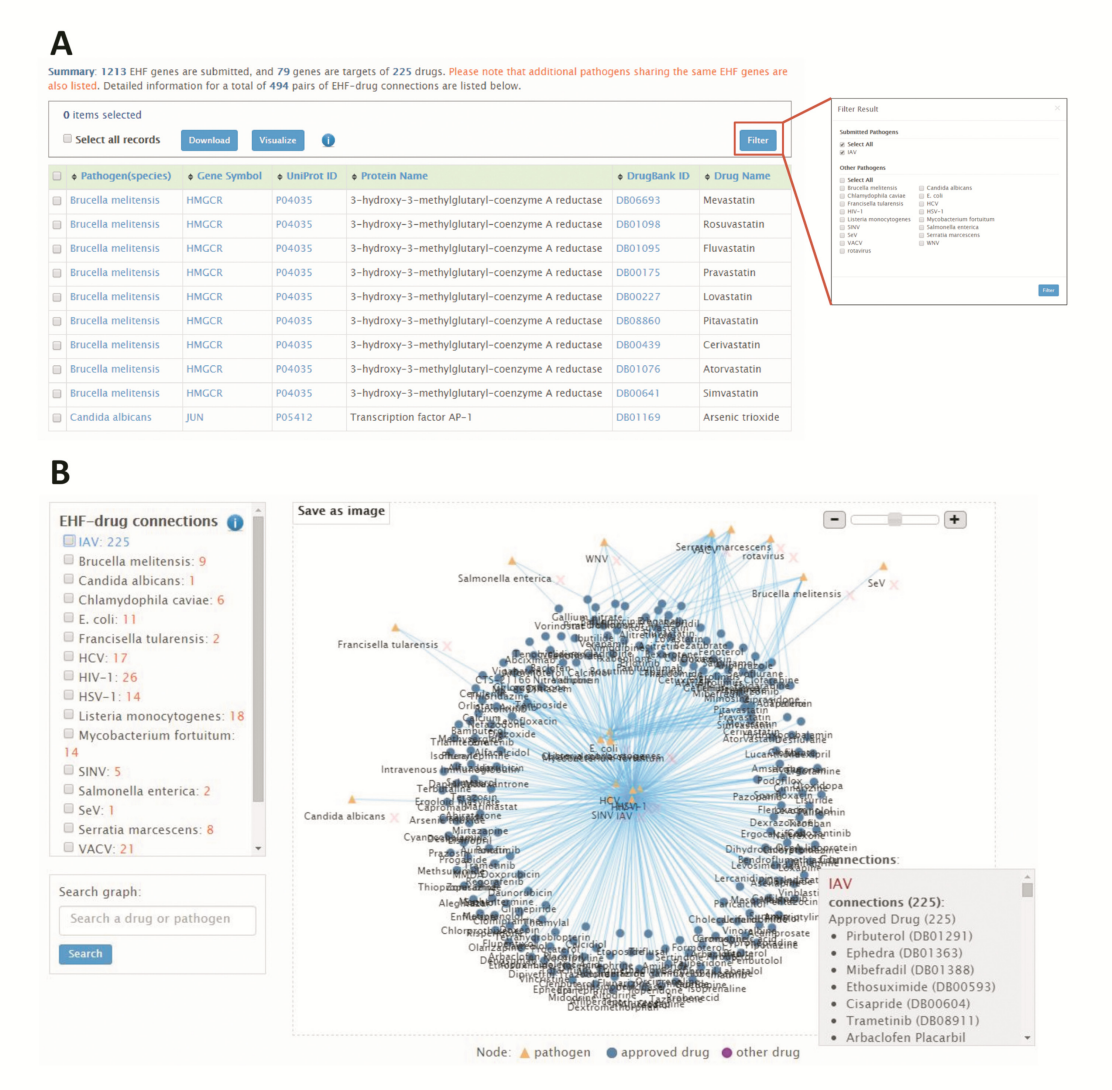
(**A**) List of Drug target analysis result for EHFs of IAV and (**B**) drug-pathogen network. Analysis results are demonstrated in a statistical summary and a list that offers detailed metadata information, and visualization of EHF-drug connection can be realized in a network presentation, in which FDA-approved drugs are highlighted, and annotation as well as external links are provided. Additional pathogens sharing the same drug targeted EHF genes are also included and detailed information on statistics and drugs is provided in independent display windows.

For the current use case, 79 out of 1213 EHF genes of IAV were identified as drug targets of 225 drugs (Figure 5A). This result indicates that in average, three drugs for one targeted EHF can be experimentally validated despite that only 6.5% EHFs of IAV can be targeted by FDA-approved drugs. We also extend the findings to those pathogens that share EHF genes with IAV, so that the potential of EHFs as drug targets for developing broad-spectrum antimicrobials can be discovered. Therefrom, a total of 494 pairs of EHF-drug connections can be observed in a network representation for 18 pathogen types that include IAV (Figure 5B), and those for individual pathogen type can be easily identified through Filter (Figure 5A), e.g. 297 pairs of EHF-drug connections for IAV. Of note, cerulenin, gallium nitrate and orlistat are highly connected drugs that show interactions with six other pathogen types besides IAV, which imply repositioning potential for developing broad-spectrum antimicrobials. Through literature investigation, we found experimental evidence that supports the antiviral activity of cerulenin against IAV (21), HIV-1 (22) and West Nile virus (23). We also found gallium nitrate is bactericidal to *M. fortuitum* (24,25), which shares EHF with IAV. Orlistat, a fatty acid synthase inhibitor, has also been validated to inhibit the replication of HIV-1 (26) and HCV (27,28) in host cells. Together, these data to some extent prove the reliability of EHFPI analysis in yielding transformative findings as in developing host-directed antimicrobial drugs.

### EHFs associating infection with disease

NHGRI GWAS Catalog (29) has provided candidate human genes adjacent to single nucleotide polymorphisms that are annotated as being associated with various diseases or disease-related intermediate phenotypes. Thus, EHFPI provides the GWAS analysis tool to permit the rapid exploration of EHF genes whose loss of function is essential to both pathogenic infection and non-infectious diseases.

For the current use case, 476 out of 1213 EHF genes of IAV were identified as GWAS candidate disease/traits genes. With further statistical evaluation, we found that EHF genes are enriched of GWAS candidate disease/traits genes (hypergeometric distribution, *P* < 0.05). We also extend the findings to those pathogens that share EHF genes with IAV, so that the potential of EHFs in indicating associations of multiple pathogenic infections and diseases/traits can be discovered. Therefrom, a total of 2167 pairs of EHF-GWAS disease/traits connections can be observed for 23 pathogen types that include IAV, and those for individual pathogen type can be easily identified through Filter, e.g. 1492 pairs of EHF-GWAS disease/traits connections for IAV (Supplementary Figure S6). Together with literature investigation, we have found support from epidemiological studies on human subjects that viral infection-disease association can be established for IAV with the diseases of alcohol dependence, bipolar disorder, breast cancer, chronic lymphocytic leukemia, coronary heart disease, Crohn's disease, inflammatory bowel disease, multiple sclerosis, schizophrenia and ulcerative colitis (Supplementary Table S3). This provides implications of shared genetic background with respect to host cellular gene dysfunction under either condition of IAV infection or diverse human disease(s).

### Conclusions from scientific use case

The workflow that was followed to explore the scientific use case enhances current bioinformatics analysis of EHF data in a more integral way that gives insight into the biological importance of EHFs beyond what general gene enrichment analysis may offer. Additional investigation will be required to determine the regulation roles of EHFs as associated with host factors at different system level. The scientific use case addressed here also underscores the ability of EHFPI to assist in generating biologically relevant hypotheses with regard to drug repositioning and discovery of infection-disease associations based on EHFs, which can then be tested experimentally.

## CONCLUSION AND PERSPECTIVES

Supported by the National Nature Science Foundation program of China, EHFPI is a specialized and integrated repository of data and analysis tools for EHFs of multiple pathogens identified from genome-wide RNAi screens. The uniqueness of EHFPI lies in (i) manually curated and categorically annotated data from extensive literatures; (ii) multi-aspect system analysis of the data contained within the system; (iii) combining the available tools to quickly perform complex analytical workflows; (iv) facilitating rapid hypothesis generation using bioinformatics methods for subsequent experimental testing; and (v) allowing graphic visualization and processing of analysis results for storage and sharing with collaborators.

EHFPI database may hold significant interest to researchers in microbiology, biomedicine and pharmaceutical industries. As a valuable resource, it will not only benefit the understanding of pathogen–host interaction and infection-disease associations, but also expedite discoveries in translational medicine and host-directed antimicrobial drugs development. The EHFPI database will continue to incorporate newly discovered EHFs in a timely manner to keep pace with this rapidly developing field. And we are encouraged to make our database more feature-rich and useful to scientists.
